# Supplementary material for: Vacancies tailoring lattice anharmonicity of Zintl-type thermoelectrics
Source: Nat Commun. 2024 Mar 23;15:2618. doi: 10.1038/s41467-024-46895-4 (PMC10960861; doi:10.1038/s41467-024-46895-4)
Supplement: Supplementary file 1 — Supplementary Information [file 41467_2024_46895_MOESM1_ESM.pdf]

Vacancies tailoring lattice anharmonicity of Zintl-type thermoelectrics

Supplementary Information

SUPPLEMENTARY NOTE 1 to 4

Supplementary Note 1 Overall isotropic ADPs analyses.

Supplementary Note 2 Heat capacity analyses.

Supplementary Note 3 Sound velocity analyses.

Supplementary Note 4 Debye-Callaway model analyses.

SUPPLEMENTARY FIG. 1 to 18

Supplementary Fig. 1 In-house XRD patterns at 300 K.

Supplementary Fig. 2 Rietveld refinements of the NPD patterns at 300 K.

Supplementary Fig. 3 Thermal and carrier transport properties of Sr(Cu,Ag,Zn)Sb compounds.

Supplementary Fig. 4 Rietveld refinement results of the NPD patterns for SrCuSb.

Supplementary Fig. 5 Rietveld refinement results of the NPD patterns for SrAgSb.

Supplementary Fig. 6 Rietveld refinement results of the NPD patterns for Sr_2ZnSb_2.

Supplementary Fig. 7 Temperature-variable lattice parameters extracted from Rietveld refinements of NPD patterns for Sr(Cu,Ag,Zn)Sb compounds.

Supplementary Fig. 8 Debye–Einstein model fitting of the experimental heat capacities.

Supplementary Fig. 9 Phonon dispersions from theoretical calculations.

Supplementary Fig. 10 Calculated phonon DOSs without considering neutron weighting effect at 300 K.

Supplementary Fig. 11 Calculated phonon DOSs at 0 K for SrCuSb, SrAgSb and at 100 K for Sr_2ZnSb_2.

Supplementary Fig. 12 Neutron-weighted phonon DOSs from inelastic neutron scattering measurements.

Supplementary Fig. 13 Charge density difference map in the (001) plane with vacancy defects.

Supplementary Fig. 14 Analysis of the X-ray photoelectron spectroscopy, XPS, data of Sr(Cu,Ag,Zn)Sb.

Supplementary Fig. 15 Anisotropic ADPs of Sr(Cu,Ag,Zn)Sb compounds.

Supplementary Fig. 16 Three- and four-phonon scattering rates in SrAgSb.

Supplementary Fig. 17 The comparison of the example Bragg peak of (110) for the Sr(Cu,Ag,Zn)Sb compounds.

Supplementary Fig. 18 Lattice thermal conductivity modeling using Debye-Callaway model.

SUPPLEMENTARY TABLE 1 to 7

Supplementary Table 1 The crystal structure information of the Sr(Cu,Ag,Zn)Sb compounds at 300 K.

Supplementary Table 2 Hall carrier concentration, Hall mobility, and band gap of the Sr(Cu,Ag,Zn)Sb compounds at 300 K.

Supplementary Table 3 Temperature-variable crystallographic parameters of the Sr(Cu,Ag,Zn)Sb compounds.

Supplementary Table 4 Parameters obtained by fitting experimental B_{ov} to the Debye–Einstein model.

Supplementary Table 5 Parameters obtained by fitting experimental heat capacity, C_P, to the Debye–Einstein model.

Supplementary Table 6 Valence state binding energy (*BE*) of different elements in Zintl compounds.

Supplementary Table 7 The crystallographic parameters of Sr(Cu,Ag,Zn)Sb refined with B_{aniso}.

Supplementary Note 1 | Overall isotropic atomic displacement parameters (ADPs) analyses

For monoatomic compound with harmonic approximation, the thermal vibration of atoms could be described with a single Debye model. However, for polyatomic compounds, the optical phonon has to been considered and it is reasonable to include both the Debye model and Einstein model[1]. The overall isotropic ADPs, B_{ov}, for the Sr(Cu,Ag,Zn)Sb compounds were analyzed with a modified Debye-Einstein model:

$$\frac{B_{\mathrm{ov}}}{8\pi^2}=\frac{A}{n(n-1)}\frac{3\hbar^2 T}{mk_{\mathrm{B}}\left(\Theta_{\mathrm{D}}^{\mathrm{ADP}}\right)^2}\left[\frac{T}{\Theta_{\mathrm{D}}}\int_0^{\Theta_{\mathrm{D}}^{\mathrm{ADP}}/T}\frac{x}{e^x-1}dx+\frac{\Theta_{\mathrm{D}}^{\mathrm{ADP}}}{4T}\right]+\frac{A}{n}\frac{\hbar^2}{2mk_{\mathrm{B}}\Theta_{\mathrm{E}}^{\mathrm{ADP}}}\coth\frac{\Theta_{\mathrm{E}}^{\mathrm{ADP}}}{2T}+d^2 \quad (1)$$

Here, the first term of the right-hand side of the equation denotes the contribution from the Debye model with Debye temperature, $\Theta_{\mathrm{D}}^{\mathrm{ADP}}$, the second term is the Einstein model with Einstein temperature, $\Theta_{\mathrm{E}}^{\mathrm{ADP}}$, and the third term d^2 comes from temperature independent disorder. T is the absolute temperature, m is average atomic mass, $\hbar$ is reduced Planck constant, A is the pre-factor of the Einstein mode (should approximate the $(n-1)$ atoms in a unit cell), and k_{B} is Boltzmann constant. With polyatomic crystals, we need to consider the dispersion relations for the acoustic (3 branches described by Debye model) and optic ($3(n-1)$ branches described by Einstein model) modes in a single Brillouin zone. Hence, parameter n (number of atoms in a unit cell) and pre-factor A should be introduced[2]. The results of the fitting are shown in Supplementary Table 4.

Supplementary Note 2 | Heat capacity analyses

We fit the experimental heat capacity, C_P, for Sr(Cu,Ag,Zn)Sb compounds with the Debye–Einstein model as follows:

$$C_P=\beta T+9R\frac{\sum_i A_i}{(n-1)}\left(\frac{T}{\Theta_{\mathrm{D}}^{\mathrm{HC}}}\right)^3\int_0^{\frac{\Theta_{\mathrm{D}}}{T}}\frac{\varepsilon^4 e^{\varepsilon}}{(e^{\varepsilon}-1)^2}d\varepsilon+3R\sum_i A_i\frac{\left(\frac{\Theta_{\mathrm{E}i}^{\mathrm{HC}}}{T}\right)^2 e^{\frac{\Theta_{\mathrm{E}i}^{\mathrm{HC}}}{T}}}{\left(e^{\frac{\Theta_{\mathrm{E}i}^{\mathrm{HC}}}{T}}-1\right)^2},\varepsilon=\frac{\Theta}{T} \quad (2)$$

In this equation, Sommerfield coefficient, β represents the electronic contribution to the specific heat. The second term gives the Debye mode contribution with $\Theta_{\mathrm{D}}^{\mathrm{HC}}$. The third term corresponds to the contribution from the Einstein mode ($\Theta_{\mathrm{E}i}^{\mathrm{HC}}$, i^{th} Einstein temperature), where A_i is the pre-factor of ith Einstein mode ($\sum_i A_i$ should approximate the n-1 atoms in a unit cell). R is the gas constant, n is the number of atoms in a unit cell. Three Einstein models are necessary to make a satisfactory fitting to the heat capacity data as illustrated in Supplementary Fig. 8. The results are summarized in Supplementary Table. 5 and Table 1 in the main text.

Supplementary Note 3 | Sound velocity analyses

Firstly, the average velocity v_{a} is calculated from the longitudinal and shear sound velocities, v_{l} and v_{s}, using the following formula[3, 4]:

$$v_{\mathrm{a}} = \left(\frac{1}{3}\left[\frac{1}{v_{\mathrm{l}}^{3}} + \frac{1}{v_{\mathrm{s}}^{3}}\right]\right)^{-\frac{1}{3}} \tag{3}$$

Then, the Debye temperature, $\Theta_{\mathrm{D}}^{\mathrm{sv}}$, is estimated from sound velocity by:

$$\Theta_{\mathrm{D}}^{\mathrm{sv}} = \frac{h}{k_{\mathrm{B}}}\left(\frac{3N}{4\pi V}\right)^{\frac{1}{3}} v_{\mathrm{a}} \tag{4}$$

where the V is the unit–cell volume, N is the number of atoms in a unit cell, k_{B} is Boltzmann parameter, and h presents the Planck constant.

Finally, the Grüneisen parameter, γ, can also be evaluated from the propagation velocities of acoustic waves as:

$$\gamma_{\mathrm{a}} = \frac{3}{2}\left(\frac{3v_{\mathrm{l}}^{2} - 4v_{\mathrm{s}}^{2}}{v_{\mathrm{l}}^{2} + 2v_{\mathrm{s}}^{2}}\right) \tag{5}$$

The results are summarized in Table 1 in the main text.

Supplementary Note 4 | Debye-Callaway model analyses

Debye-Callaway model was used to analyze the lattice thermal conductivity with phonon relaxation time contributed by different scattering processes:

$$\kappa_{\mathrm{lat}} = \int_{0}^{\omega_{\mathrm{D}}} \kappa_{\mathrm{S}}(\omega)\, d\omega = \frac{k_{\mathrm{B}}}{2\pi^{2} v}\left(\frac{k_{\mathrm{B}}T}{\hbar}\right)^{3} \int_{0}^{\theta_{\mathrm{D}}/T} \tau \frac{x^{4} e^{x}}{(e^{x}-1)^{2}} dx \tag{6}$$

where k_{B}, v, $\hbar$, T, θ_{D}, and τ are the Boltzmann constant, average sound velocity, reduced Planck constant, absolute temperature, Debye temperature, and phonon relaxation time, respectively. x is the reduced phonon frequency $x = \hbar\omega/k_{\mathrm{B}}T$. The phonon relaxation time τ can be obtained by considering the contributions from different scattering processes:

$$\tau^{-1} = \tau_{\mathrm{U}}^{-1} + \tau_{\mathrm{N}}^{-1} + \tau_{\mathrm{B}}^{-1} + \tau_{\mathrm{V}}^{-1} \tag{7}$$

where τ_{U}, τ_{N}, τ_{B}, and τ_{V} refer to the phonon relaxation time for Umklapp scattering (U), normal scattering (N), boundary scattering (B), and vacancy-related scattering (V), respectively. In this analysis, we set SrAgSb as pristine material. The Umklapp scattering, normal scattering, and boundary scattering are considered in SrAgSb. For the Sr_2ZnSb_2, the Umklapp scattering, normal scattering, boundary scattering, and vacancy-related scattering are considered. Here, the vacancy-related scatterings include the enhanced phonon anharmonicity scatterings and phonon-point defect scatterings. The results are shown in Supplementary Fig. 18.

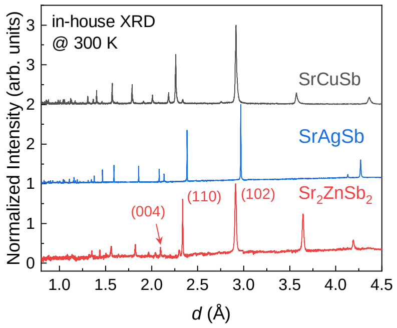

Supplementary Fig. 1 | In-house XRD patterns at 300 K. The patterns are presented in the plane distance, d, space. All three samples can be indexed with the hexagonal *P*6₃/*mmc* structure but without discernible impurities. The (102), (110) and (004) Bragg peaks are labeled.

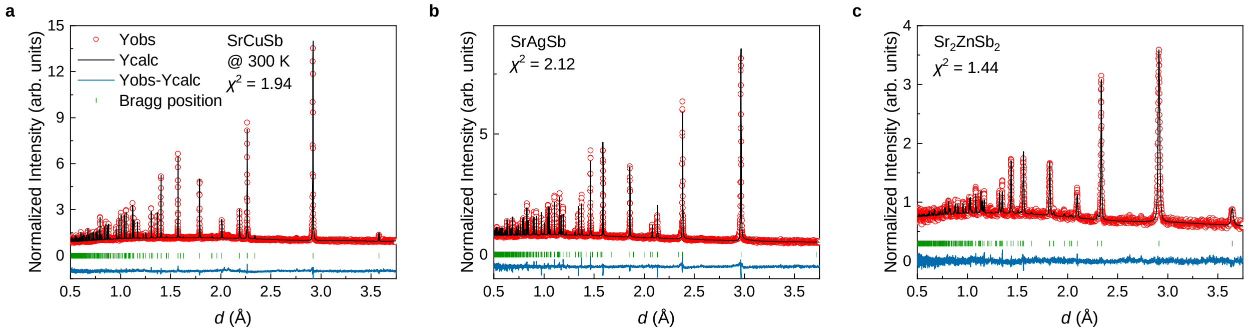

Supplementary Fig. 2 | Rietveld refinements of the neutron powder diffraction (NPD) patterns collected on the SuperHRPD diffractometer at 300 K. a SrCuSb, **b** SrAgSb, and **c** Sr_2ZnSb_2.

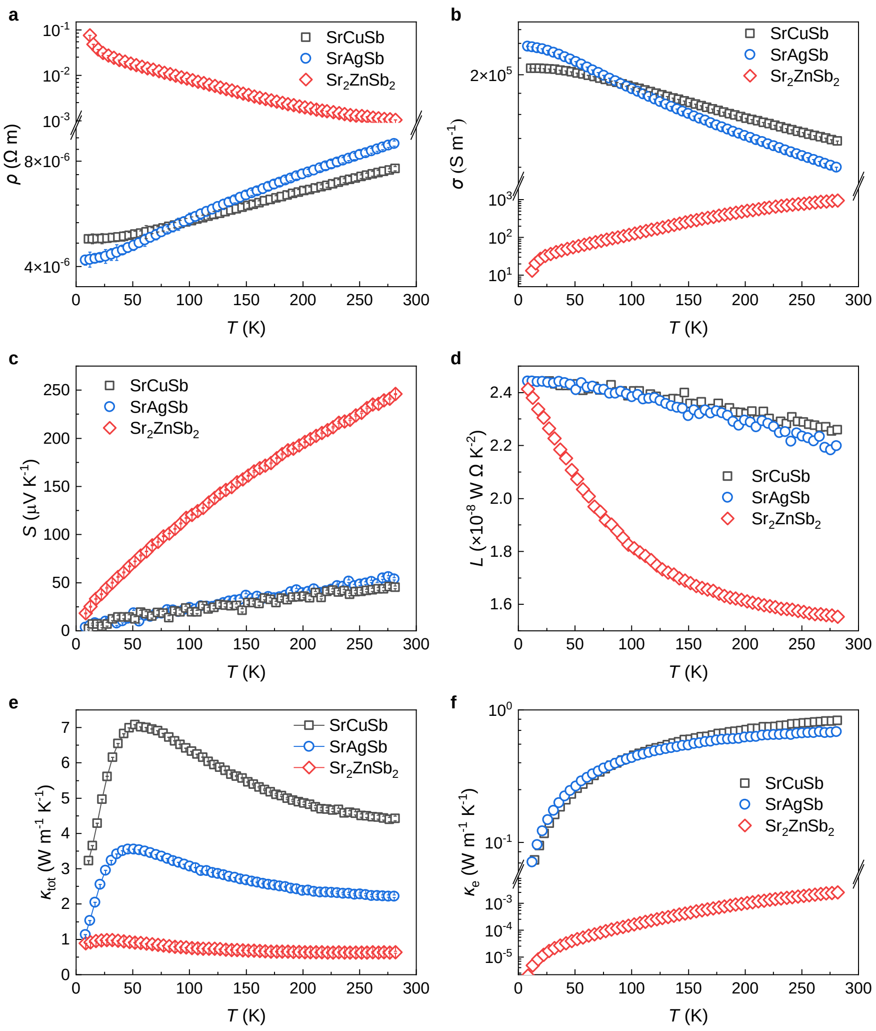

Supplementary Fig. 3 | Thermal and carrier transport properties of Sr(Cu,Ag,Zn)Sb compounds. a Resistivity, ρ. **b** Carrier conductivity, σ. **c** Seebeck coefficient, S. **d** Lorenz number, L. **e** Temperature variable total thermal conductivity, κ_{tot}. **f** Electrical thermal conductivity, κ_{e}. It is noted that the Sr_2ZnSb_2 sample has a much lower electrical conductivity in (**b**). According to the Hall carrier concentration, mobility, and band gap measurements (see Supplementary Table 2), this poor electrical conductivity is attributed to the much lower Hall carrier concentration and much smaller mobility. The error bars in (**a**-**c**,**e**) come from the data measurements using PPMS.

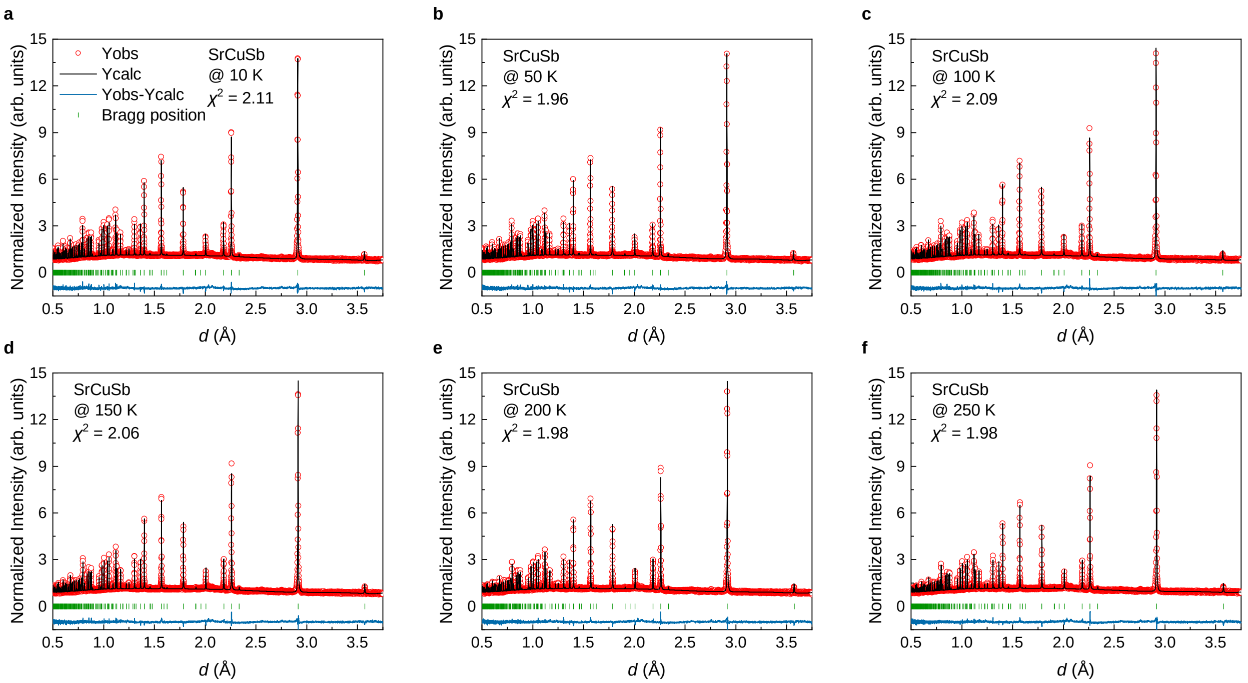

Supplementary Fig. 4 | Rietveld refinement results of the NPD patterns (SuperHRPD) for SrCuSb (**a-f**) at 10, 50, 100, 150, 200, 250 K, respectively.

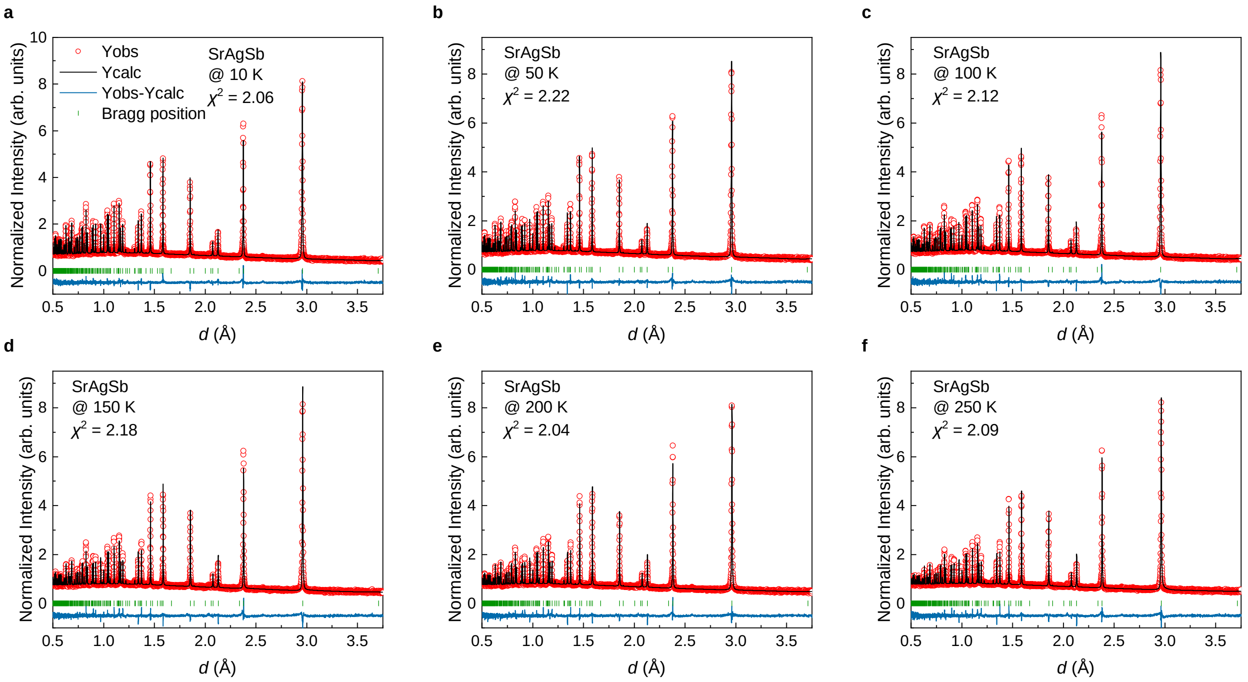

Supplementary Fig. 5 | Rietveld refinement results of the NPD patterns (SuperHRPD) for SrAgSb (**a-f**) at 10, 50, 100, 150, 200, 250 K, respectively.

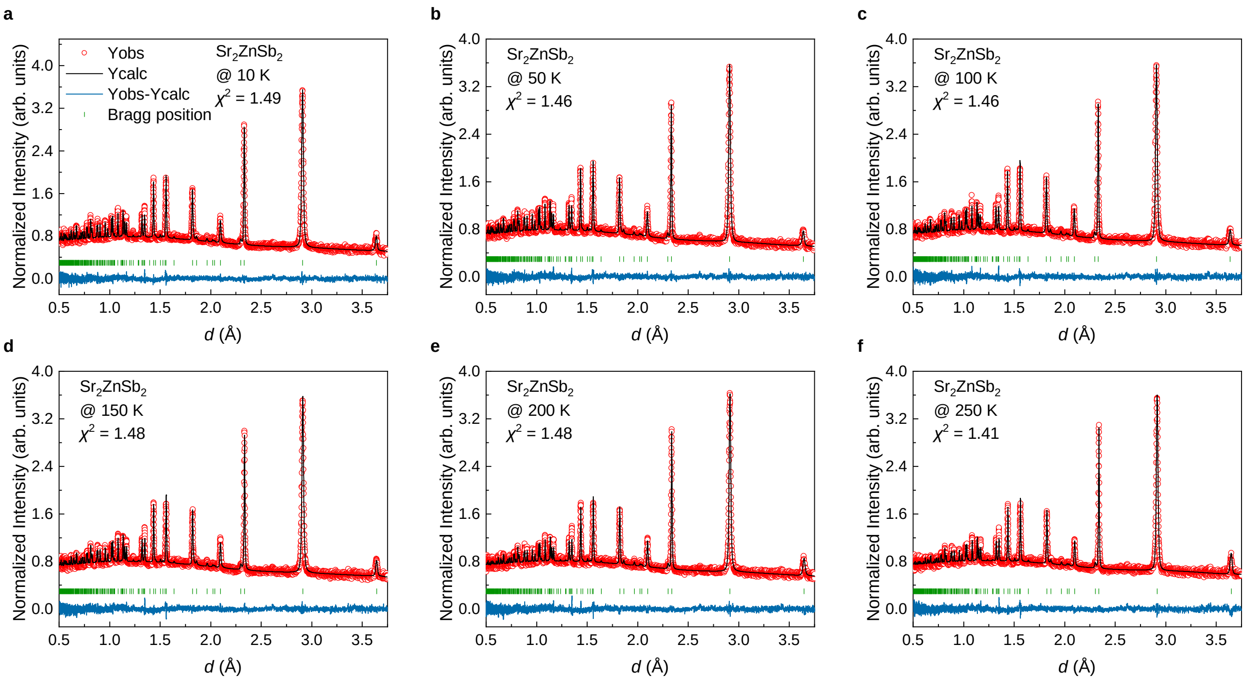

Supplementary Fig. 6 | Rietveld refinement results of the NPD patterns (SuperHRPD) for Sr_2ZnSb_2 (a-f) at 10, 50, 100, 150, 200, 250 K, respectively.

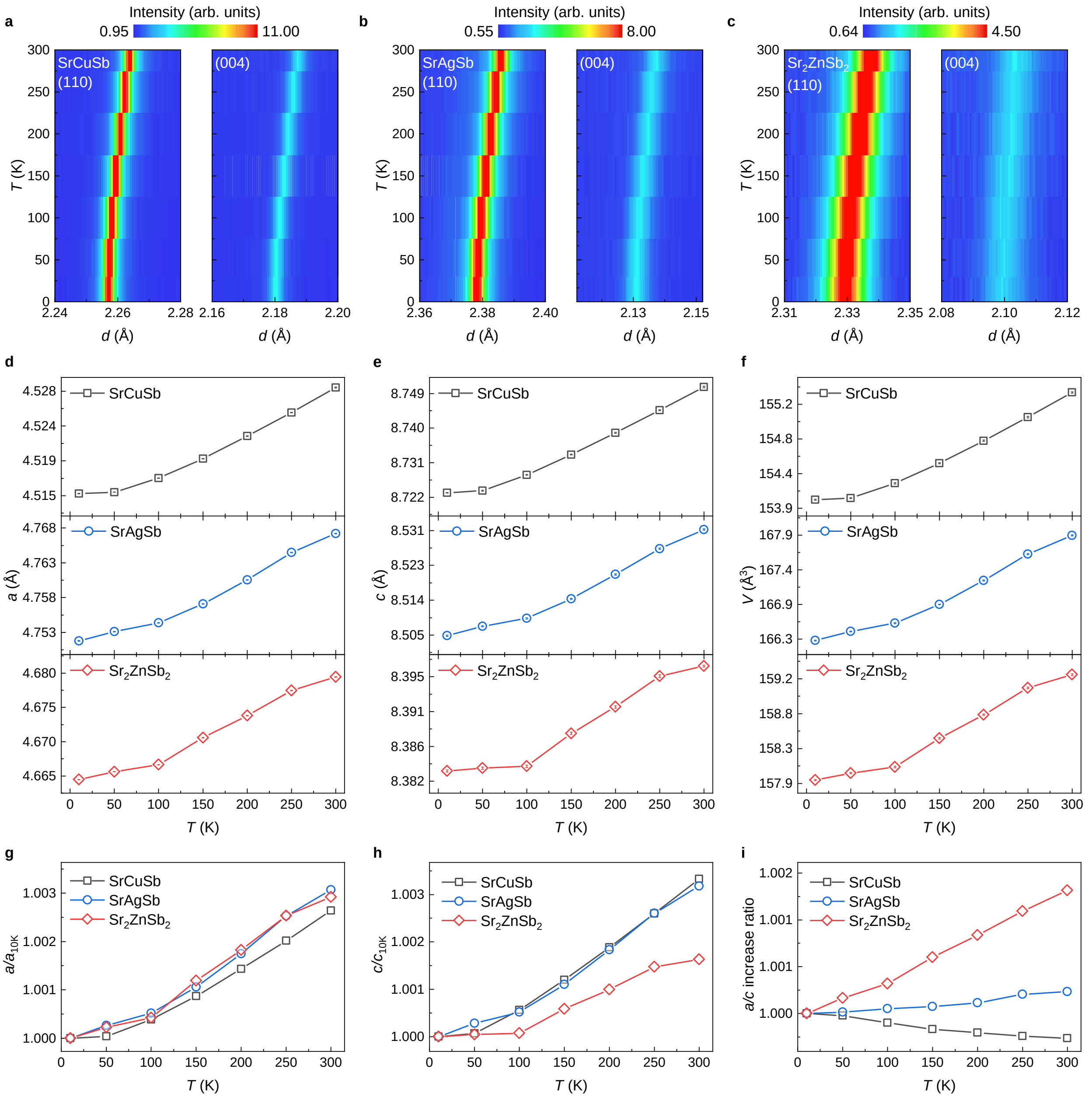

Supplementary Fig. 7 | Temperature-variable lattice parameters extracted from Rietveld refinements of NPD patterns collected on the SuperHRPD diffractometer. **a**-**c** Variation of the (110) and (004) Bragg peaks with temperature for (**a**) SrCuSb, (**b**) SrAgSb, and (**c**) Sr_2ZnSb_2, respectively. **d**-**f** Temperature dependencies of lattice parameters (**d**) a, (**e**) c, and (**f**) unit cell volume, V. Details are summarized in Supplementary Table 3. The error bars represent the standard deviation from the Rietveld refinements. **g**-**i** Increase ratio of lattice parameters (**g**) a, (**h**) c, and (**i**) a/c ratio with respect to 10 K value.

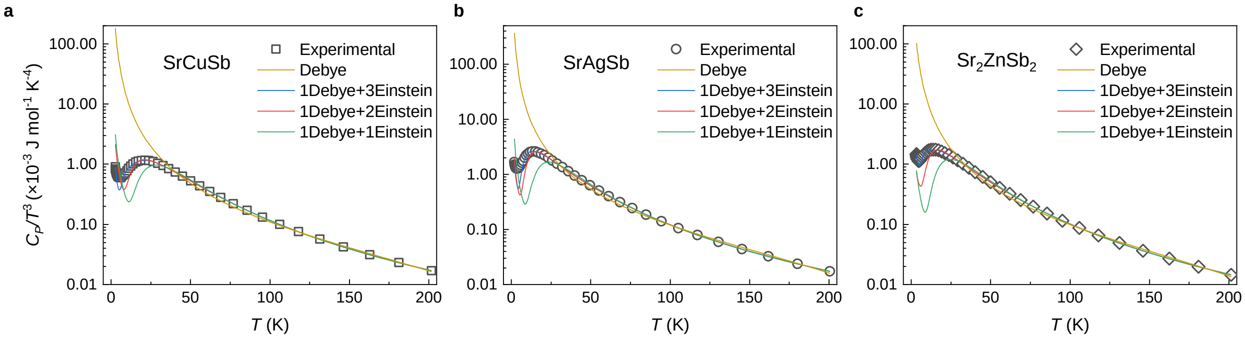

Supplementary Fig. 8 | Debye–Einstein model fitting of the experimental heat capacities. a-c C_P/T^3 vs. T plots of the comparison between experimental data and fitted curves by using 1 Debye model plus 0, 1, 2 or 3 Einstein modes for the samples (**a**) SrCuSb, (**b**) SrAgSb, and (**c**) Sr_2ZnSb_2.

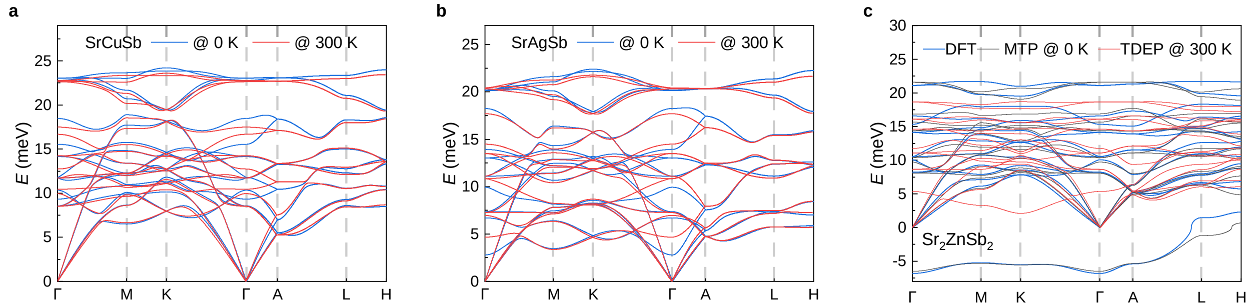

Supplementary Fig. 9 | Phonon dispersions from theoretical calculations. a-c The phonon dispersions at 0 K and 300 K for (**a**) SrCuSb and (**b**) SrAgSb, and at (**c**) 0 K and 300 K for Sr_2ZnSb_2 with experimental lattice parameters. The temperature-renormalized phonon dispersions were calculated with the temperature-dependent effective potential, TDEP, method. It is noted that the phonon DOS for Sr_2ZnSb_2 with the general special quasi-random structure, SQS, at 300 K in the main manuscript is calculated from equilibrium molecular dynamics, EMD, simulations. The energy range of the renormalized phonons from EMD simulation gives a closer result to the experimental data, as the SQS structure captures the disordered Zn vacancies state and more resembles to the real defective samples. The phonon dispersions of Sr_2ZnSb_2 calculated with the primitive unit cell at 0 K from DFT and MTP (moment tensor potential) in (**c**) give imaginary phonon frequencies, which are stabilized with the TDEP method at 300 K. Although the phonon energy range for Sr_2ZnSb_2 at 300 K from the TDEP method is lower than the EMD simulations and experimental INS results (Fig. 3), it captures the overall phonon softening observed from the experiment.

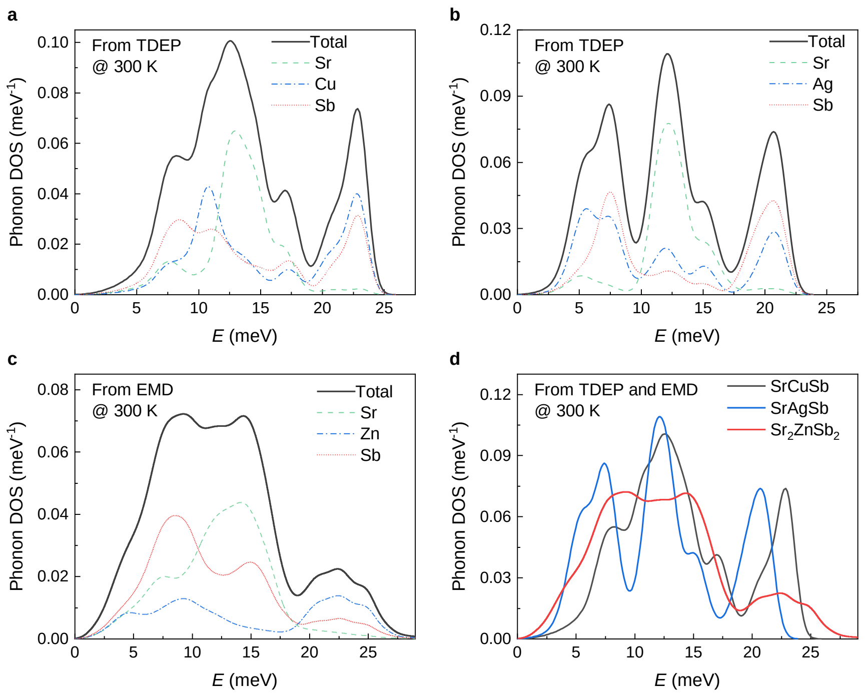

Supplementary Fig. 10 | Calculated phonon density of states (DOSs) without considering neutron weighting effect (neutron cross-sections) at 300 K. a-**c** Total and partial phonon DOS, without considering difference of atomic mass, m, and neutron scattering cross section, σ, between different elements, for Sr(Cu,Ag,Zn)Sb obtained from TDEP and EMD simulations. The values of σ/m for Sr, Cu, Ag, Zn, Sb are 0.07133, 0.12636, 0.04626, 0.06318, 0.03203 barn amu^{-1} (1 barn = 1×10^{-24} cm^2, amu represents atomic mass unit), respectively. **d** Comparison of calculated total phonon DOSs.

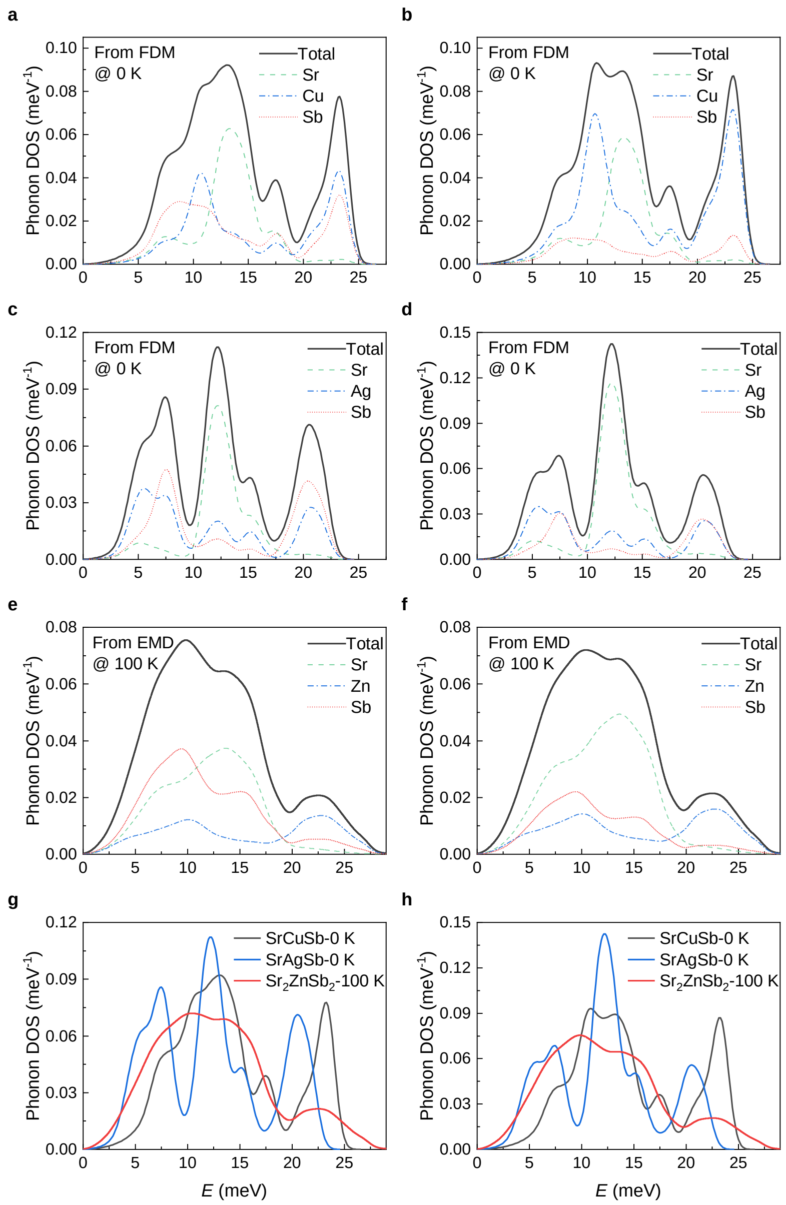

Supplementary Fig. 11 | Calculated phonon DOSs at 0 K for SrCuSb, SrAgSb and at 100 K for Sr_2ZnSb_2. a-d Total and partial phonon DOS at 0 K for SrCuSb and SrAgSb from the finite displacement method, FDM, (**a**,**c**) without and (**b**,**d**) with neutron weighting. **e**,**f** Total and partial phonon DOSs at 100 K for Sr_2ZnSb_2 from EMD simulation (**e**) without and (**f**) with neutron weighting. **g**,**h** Comparison of calculated total phonon DOSs (**g**) without and (**h**) with neutron-weighting for the Sr(Cu, Ag, Zn)Sb compounds.

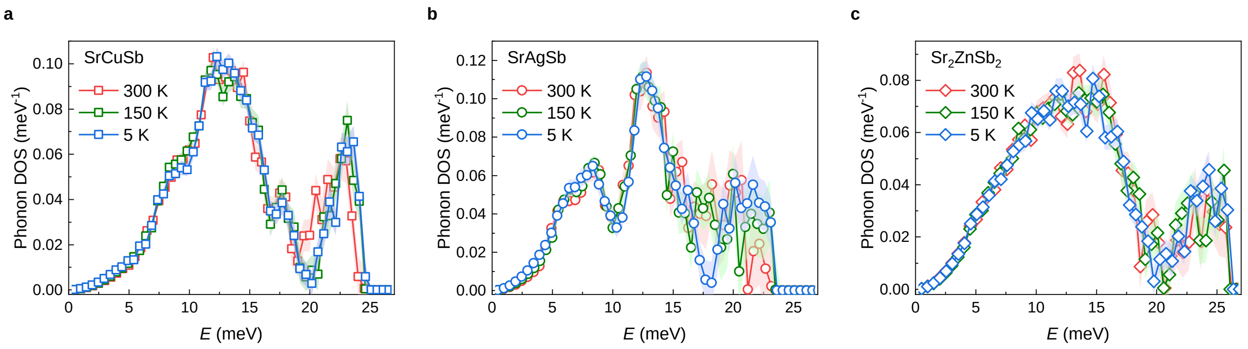

Supplementary Fig. 12 | Neutron-weighted phonon density of states (DOSs) from inelastic neutron scattering measurements. a-c Phonon DOSs at 5, 150 and 300 K are collected for (**a**) SrCuSb, (**b**) SrAgSb, and (**c**) Sr_2ZnSb_2, respectively. Error bars represent one standard deviation from the phonon DOS calculation using GetDOS[5].

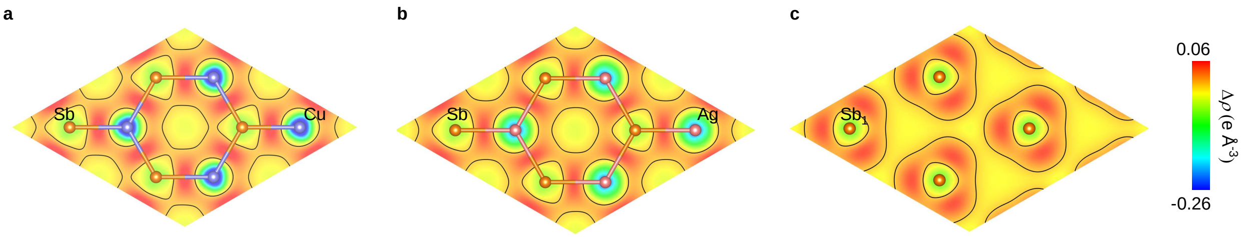

Supplementary Fig. 13 | Charge density difference map in the (001) plane with Zn vacancy defects. a-c Comparison of the [CuSb], [AgSb] and Zn vacancy planes for (**a**) SrCuSb, (**b**) SrAgSb, and (**c**) Sr_2ZnSb_2, respectively.

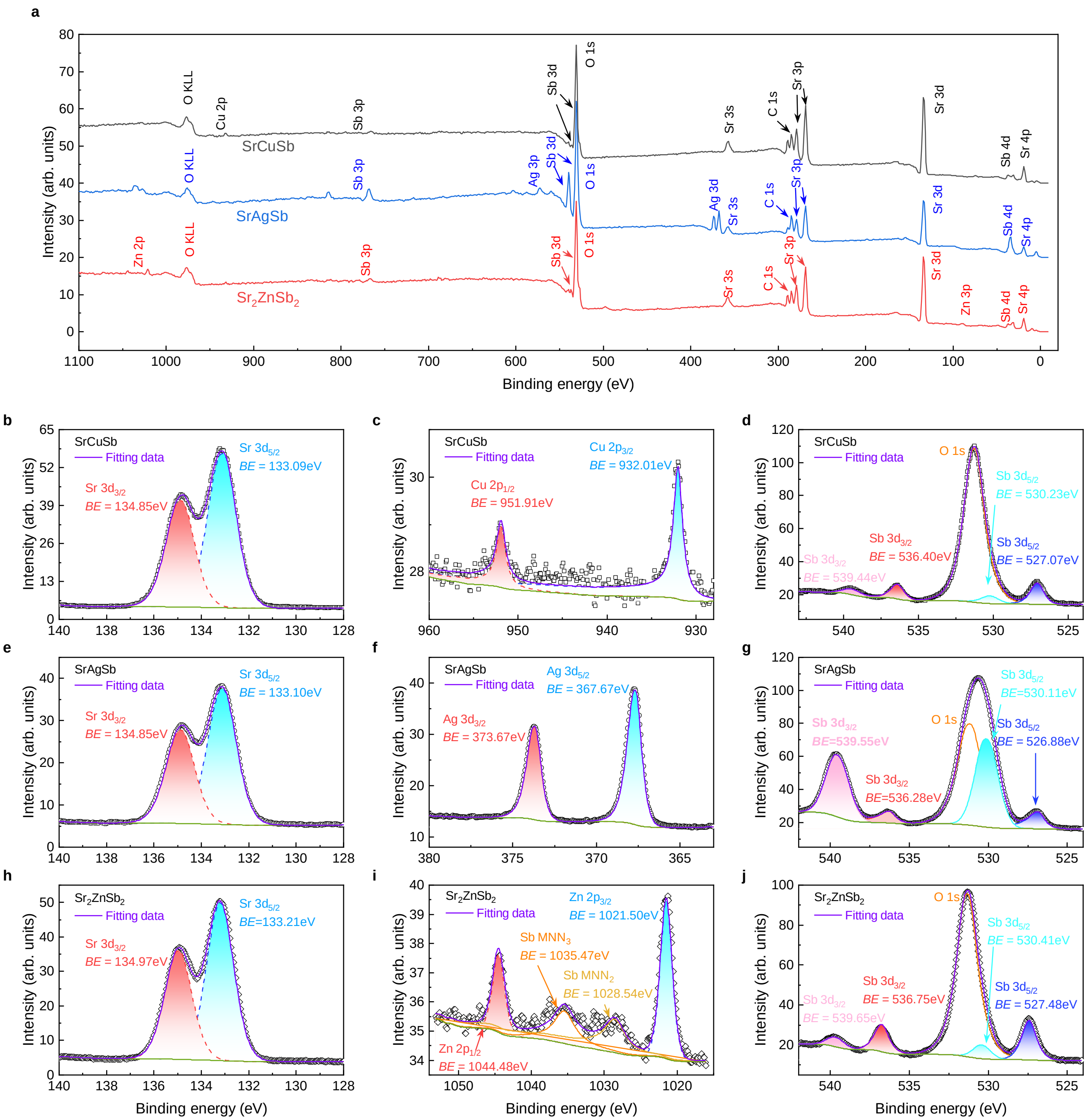

Supplementary Fig. 14 | Analysis of the X-ray photoelectron spectroscopy, XPS, data of Sr(Cu,Ag,Zn)Sb. **a** XPS Survey (fast wide-scan) chemical qualification of Sr(Cu,Ag,Zn)Sb. **b**-**d** The XPS spectrum and fitting results of Sr 3d, Cu 2p, and Sb 3d from the SrCuSb powder sample. **e**-**g**, The XPS spectrum and fitting results of Sr 3d, Ag 2p, and Sb 3d from the SrAgSb powder sample. **h**-**j** The XPS spectrum and fitting results of Sr 3d, Zn 2p, and Sb 3d from the Sr_2ZnSb_2 powder sample. *BE* represents binding energy. Auger signal of Sb named as MNN_2 (*BE* = 1028.54 eV), MNN_3 (*BE* = 1028.54 eV) was detected in (**i**). The results are summarized in Supplementary Table 6.

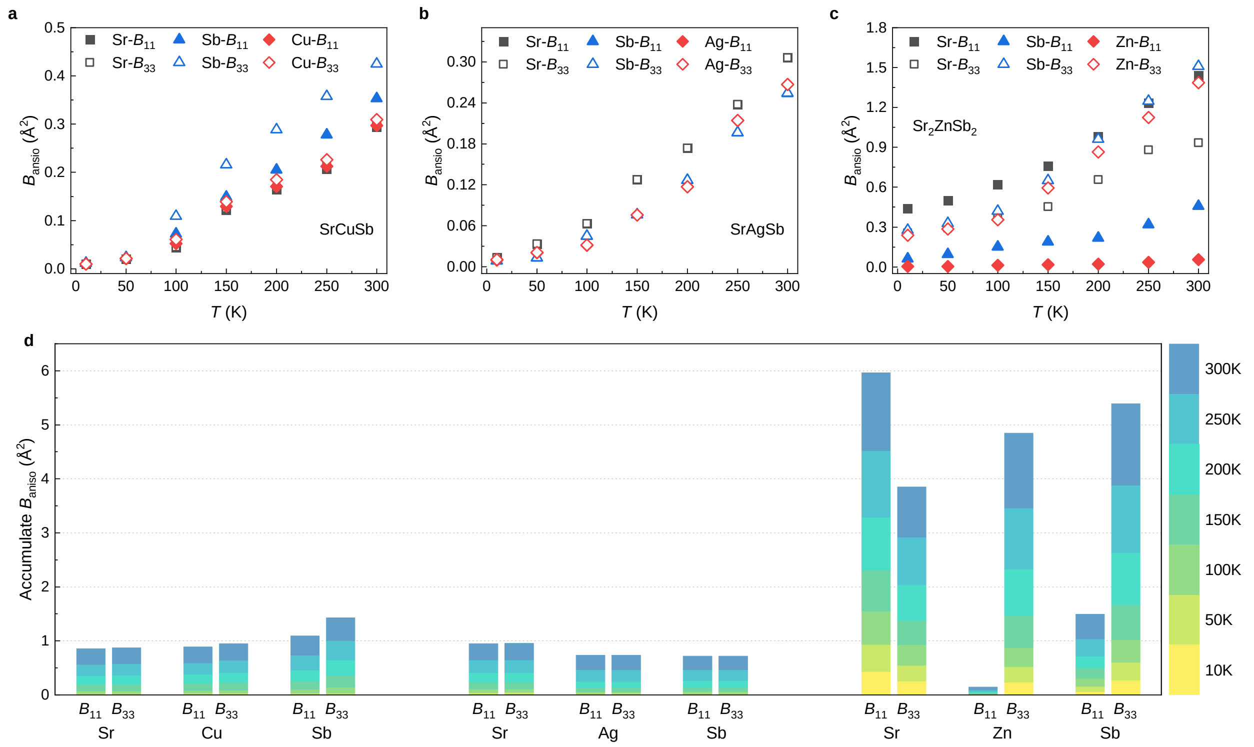

Supplementary Fig. 15 | Anisotropic ADPs of Sr(Cu,Ag,Zn)Sb compounds. a-c Temperature variable anisotropic ADPs for each atom in (**a**) SrCuSb, (**b**) SrAgSb, and (**c**) Sr_2ZnSb_2 compounds, respectively. **d** Anisotropic ADPs shown in histogram format for easy comparison.

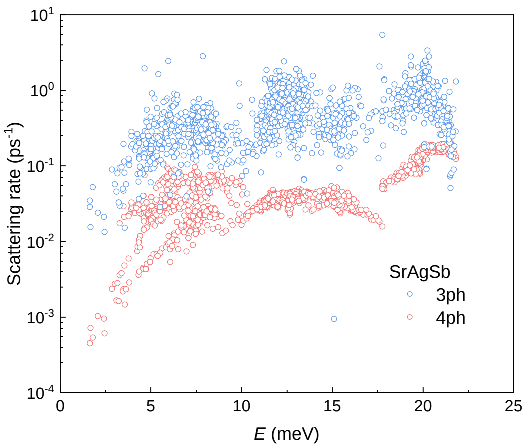

Supplementary Fig. 16 | Three- and four-phonon scattering rates in SrAgSb at 300 K. The phonon scattering rates were calculated with the renormalized interatomic force constants using the ShengBTE package[6]. We follow the same protocol as our previous work to extract the third- and fourth-order constants, denoting as 3ph and 4ph in the figure, respectively[7]. It is noted that the experimental lattices are adopted to generate the reference training structures. Similar information for SrCuSb and Sr_2ZnSb_2 has been provided in the previous work[7]. All three compounds show a larger three-phonon scatterings than four-phonon scatterings. For SrAgSb, there is a strong enhancement on the scattering rate between ~4 to ~9 meV in comparison with SrCuSb, which should be caused by the strong softening of the low-lying optical phonon due to the heavier atomic mass of Ag. However, Sr_2ZnSb_2 presents large increases in both three- and four-phonon scattering rates over the whole energy range in comparison with both SrCuSb and SrAgSb, agreeing well with results from neutron scattering data and atomic bonding analyses.

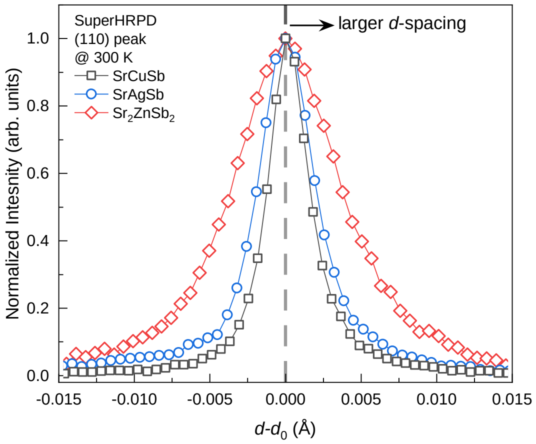

Supplementary Fig. 17 | The comparison of the example Bragg peak of (110) for the Sr(Cu,Ag,Zn)Sb compounds. The data were collected at 300 K on the SuperHRPD high resolution neutron diffractometer at J-PARC in Japan. As shown in this, the example (110) Bragg peak of the Sr_2ZnSb_2 compound has a much larger full width at half maximum, FWHM, demonstrating that the vacancies in Sr_2ZnSb_2 also present a strong strain field. This behavior confirms that the vacancies in Sr_2ZnSb_2 play a phonon-point defect scattering role rather than a phonon-vacancy scattering role as in SnTe[8-10]. In SeTe, phonon-vacancy scattering physical picture considers that the vacancy only induces a mass-fluctuations in the matrix but without strain fluctuation, while point defect scattering contain both fluctuations.

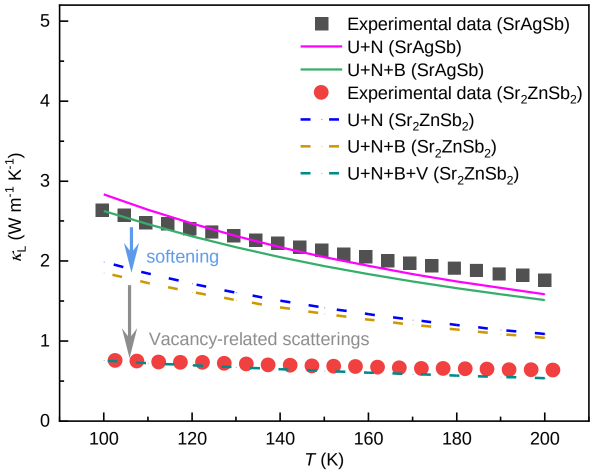

Supplementary Fig. 18 | Lattice thermal conductivity modeling using Debye-Callaway model. The SrAgSb is taken as the pristine materials, and it is clear that the vacancy-related scatterings make large contribution to the suppression of the lattice thermal conductivity in the vacancy-defective Sr_2ZnSb_2. The phonon relaxation time contributed from the Umklapp scattering (U), normal scattering (N), boundary scattering (B), and vacancy-related scattering (V) are considered. Here, the vacancy-related scatterings include the enhanced phonon anharmonicity scatterings and phonon-point defect scatterings. More details about the fitting are given in Supplementary Note 4.

Supplementary Table 1 | The crystal structure information of the Sr(Cu,Ag,Zn)Sb compounds.

**SrCuSb @ 300 K**<br>**Space group:** *P*6_3/*mmc*<br>**Unit cell (Å):** a = 4.5284(6), c = 8.7510(19)<br>χ^2**:** 1.94						
**Atom**	**Site**	**Symmetry**	*x*	*y*	*z*	**Occupancy**
Sr	2*a*	-3*m*.	0	0	0	1
Sb	2*c*	-6*m*2	1/3	2/3	1/4	1
Cu	2*d*	-6*m*2	1/3	2/3	3/4	1
**SrAgSb @ 300 K**<br>**Space group:** *P*6_3/*mmc*<br>**Unit cell (Å):** a = 4.7676(5), c = 8.5315(16)<br>χ^2**:** 1.99						
**Atom**	**Site**	**Symmetry**	*x*	*y*	*z*	**Occupancy**
Sr	2*a*	-3*m*.	0	0	0	1
Sb	2*c*	-6*m*2	1/3	2/3	1/4	1
Ag	2*d*	-6*m*2	1/3	2/3	3/4	1
**Sr_2ZnSb_2 @ 300 K**<br>**Space group:** *P*6_3/*mmc*<br>**Unit cell (Å):** a = 4.6795(4), c = 8.3966(12)<br>χ^2**:** 1.34						
**Atom**	**Site**	**Symmetry**	*x*	*y*	*z*	**Occupancy**
Sr	2*a*	-3*m*.	0	0	0	1
Sb	2*c*	-6*m*2	1/3	2/3	1/4	1
Zn	2*d*	-6*m*2	1/3	2/3	3/4	0.5

The data were derived from the Rietveld refinements of the NPD patterns at 300 K in Supplementary Fig. 2.

Supplementary Table 2 | Hall carrier concentration, Hall mobility, and electrical band gap of the Sr(Cu,Ag,Zn)Sb compounds at 300 K.

Sample	Hall carrier concentration (cm^{-3})	Hall mobility ($cm^2 V^{-1} s^{-1}$)	Band gap (eV)
SrCuSb	1.2×10^{20}	158	0.13
SrAgSb	4.5×10^{19}	254	0.17
Sr_2ZnSb_2	4.3×10^{18}	21	0.12

The band gaps were estimated from the analysis of optical spectra.

Supplementary Table 3 | The crystallographic parameters of the Sr(Cu,Ag,Zn)Sb compounds.

**SrCuSb**						
T (K)	a (Å)	c (Å)	V (Å^3)	R_{wp} (%)	χ^2	B_{ov} (Å^2)
10	4.5153(2)	8.7228(9)	154.011(7)	3.97	2.11	0.00995(8)
50	4.5154(2)	8.7234(15)	154.032(6)	3.82	1.96	0.02018(5)
100	4.5172(3)	8.7276(11)	154.225(9)	3.94	2.09	0.07197(5)
150	4.5196(3)	8.7329(12)	154.485(8)	3.90	2.06	0.17152(6)
200	4.5224(4)	8.7387(13)	154.778(9)	3.82	1.98	0.24672(8)
250	4.5253(4)	8.7448(13)	155.084(9)	3.79	1.98	0.33581(7)
300	4.5284(6)	8.7510(19)	155.405(8)	3.74	1.94	0.43065(9)
**SrAgSb**						
T (K)	a (Å)	c (Å)	V (Å^3)	R_{wp} (%)	χ^2	B_{ov} (Å^2)
10	4.7515(5)	8.5053(16)	166.298(5)	4.71	2.06	0.00359(1)
50	4.7529(4)	8.5076(14)	166.440(7)	4.89	2.22	0.03415(5)
100	4.7542(4)	8.5095(15)	166.570(8)	4.77	2.12	0.04714(6)
150	4.7571(5)	8.5143(15)	166.862(4)	4.82	2.18	0.12707(7)
200	4.7607(4)	8.5204(15)	167.234(8)	4.64	2.04	0.20632(7)
250	4.7648(5)	8.5267(16)	167.646(9)	4.68	2.09	0.34496(9)
300	4.7676(5)	8.5315(19)	167.938(8)	4.69	2.12	0.44113(10)
**Sr_2ZnSb_2**						
T (K)	a (Å)	c (Å)	V (Å^3)	R_{wp} (%)	χ^2	B_{ov} (Å^2)
10	4.6645(3)	8.3833(14)	157.961(7)	4.01	1.49	0.34096(15)
50	4.6656(3)	8.3837(14)	158.046(8)	3.97	1.46	0.40317(16)
100	4.6666(3)	8.3839(15)	158.119(9)	3.96	1.46	0.36796(17)
150	4.6706(3)	8.3881(15)	158.462(10)	3.96	1.48	0.56536(15)
200	4.6738(4)	8.3914(15)	158.748(8)	3.95	1.48	0.61805(20)
250	4.6775(4)	8.3953(16)	159.069(10)	3.83	1.41	0.80787(20)
300	4.6795(4)	8.3966(12)	159.229(12)	3.86	1.44	0.96006(22)

The data were derived from the Rietveld refinements of the NPD patterns in Supplementary Figs. 2,4-6.

Supplementary Table 4 | Parameters obtained by fitting experimental $\boldsymbol{B}_{\mathbf{ov}}$ to the Debye–Einstein model.

Parameter	SrCuSb	SrAgSb	Sr_2ZnSb_2
d^2	-0.00166(3)	-0.00147(14)	0.00211(15)
m	90.97	105.75	96.83
$\Theta_{\mathrm{D}}^{\mathrm{ADP}}$ (K)	279(3)	248(7)	218(8)
$\Theta_{\mathrm{E}}^{\mathrm{ADP}}$ (K)	148(2)	142(3)	126(3)
A	4.86(4)	4.78(6)	3.96(6)
n	6	6	5
R^2	0.99825	0.96819	0.955248
Reduced Chi-Square	8.84440×10^{-9}	1.43294×10^{-7}	4.75799×10^{-7}

m: averaged atomic mass, n: number of atoms in a unit cell, A: pre-factor of the Einstein mode. Refer Supplementary Note 1 and Fig. 2a-c in main text for more information.

Supplementary Table 5 | Parameters obtained by fitting experimental heat capacity.

**Parameter**	**SrCuSb**	**SrAgSb**	**Sr_2ZnSb_2**
β (10^{-3} J mol^{-1} K^{-2})	0.00550(2)	0.00586(4)	0.01372(4)
$\Theta_{\mathrm{D}}^{\mathrm{HC}}$ (K)	269.79(5)	235.32(8)	217.26(8)
A_1	0.25(7)	0.73(10)	0.32(12)
$\Theta_{\mathrm{E1}}^{\mathrm{HC}}$ (K)	58.94(5)	56.47(2)	51.48(5)
A_2	2.63(9)	2.42(7)	1.87(6)
$\Theta_{\mathrm{E2}}^{\mathrm{HC}}$ (K)	124.41(4)	111.89(4)	108.59(5)
A_3	2.03(1)	1.77(1)	1.73(3)
$\Theta_{\mathrm{E3}}^{\mathrm{HC}}$ (K)	222.58(4)	218.30(5)	210.10(5)
N	6	6	5
R^2	0.999996	0.999995	0.99999
Reduced Chi-Square	0.010051	0.015199	0.019474

The data were extracted by fitting experimental heat capacity, C_P, to the Debye–Einstein model. Refer Supplementary Note 2, Supplementary Fig. 8 and Fig. 2d-g in main text for more information.

Supplementary Table 6 | Valence state binding energy (*BE*) of different elements in Zintl compounds.

Element		*BE* (eV)		
		**SrCuSb**	**SrAgSb**	**Sr_2ZnSb_2**
Sr	$3d_{5/2}$	133.09	133.10	133.21
	$3d_{3/2}$	134.85	134.85	134.97
Cu	$2p_{3/2}$	932.01		
	$2p_{1/2}$	951.91		
Ag	$3d_{5/2}$		367.67	
	$3d_{3/2}$		373.67	
Zn	$2p_{3/2}$			1021.50
	$2p_{1/2}$			1044.48

The data are extracted from the refinement of the powder XPS data in Supplementary Fig. 14.

Supplementary Table 7 | The crystallographic parameters of Sr(Cu,Ag,Zn)Sb refined with B_{aniso}.

**SrCuSb**						
T (K)	R_{wp} (%)	χ^2	Atom	B_{eq} (Å^2)	$B_{11}=B_{22}=2B_{12}$ (Å^2)	B_{33} (Å^2)
10	3.95	2.09	Sr	0.00994(6)	0.00994(3)	0.00995(12)
			Cu	0.00995(7)	0.00995(4)	0.00995(13)
			Sb	0.01131(6)	0.01131(3)	0.01132(11)
50	3.79	1.93	Sr	0.02018(6)	0.02015(2)	0.02023(14)
			Cu	0.02104(7)	0.02099(2)	0.02115(16)
			Sb	0.02327(7)	0.02319(4)	0.02341(12)
100	3.86	2.01	Sr	0.04437(7)	0.04426(5)	0.04458(10)
			Cu	0.05535(5)	0.05257(3)	0.06092(8)
			Sb	0.08460(3)	0.07253(2)	0.10873(6)
150	3.83	1.99	Sr	0.12255(6)	0.12131(4)	0.12504(9)
			Cu	0.13279(9)	0.12957(7)	0.13921(13)
			Sb	0.17139(5)	0.14928(2)	0.21558(11)
200	3.71	1.88	Sr	0.16572(5)	0.16482(4)	0.16752(7)
			Cu	0.17551(4)	0.17092(3)	0.18469(6)
			Sb	0.23312(6)	0.20540(4)	0.28856(9)
250	3.72	1.91	Sr	0.20887(4)	0.20699(3)	0.21263(6)
			Cu	0.21723(4)	0.21274(3)	0.22621(7)
			Sb	0.30442(6)	0.27796(4)	0.35733(10)
300	3.63	1.83	Sr	0.29454(9)	0.29375(8)	0.29612(13)
			Cu	0.30111(10)	0.29682(8)	0.30969(13)
			Sb	0.37711(12)	0.35318(10)	0.42497(16)

**SrAgSb**						
T (K)	R_{wp} (%)	χ^2	Atom	B_{eq} (Å^2)	$B_{11}=B_{22}=2B_{12}$ (Å^2)	B_{33} (Å^2)
10	4.44	1.83	Sr	0.01346(5)	0.01345(2)	0.01349(12)
			Ag	0.00985(6)	0.00950(5)	0.01055(7)
			Sb	0.00889(7)	0.00889(5)	0.00890(10)

50	4.58	1.95	Sr	0.03334(6)	0.03332(2)	0.03337(14)
			Ag	0.02064(6)	0.02061(5)	0.02069(9)
			Sb	0.01302(9)	0.01300(7)	0.01304(13)
100	4.68	2.04	Sr	0.06297 (2)	0.06295 (2)	0.06301 (3)
			Ag	0.03159 (4)	0.03156 (3)	0.03166 (6)
			Sb	0.04484 (5)	0.04481 (3)	0.04492 (8)
150	4.69	2.07	Sr	0.12748(6)	0.12748(5)	0.12748(7)
			Ag	0.07555(3)	0.07555(3)	0.07556(4)
			Sb	0.07620(4)	0.07620(3)	0.07621(7)
200	4.48	1.90	Sr	0.17387(13)	0.17387(11)	0.17387(19)
			Ag	0.11719(3)	0.11719(3)	0.11720(4)
			Sb	0.12689(9)	0.12689(7)	0.12690(12)
250	4.55	1.98	Sr	0.23763(12)	0.23763(10)	0.23764(15)
			Ag	0.21417(3)	0.21417(3)	0.21418(5)
			Sb	0.19627(11)	0.19627(9)	0.19628(16)
300	4.54	1.99	Sr	0.30597(13)	0.30592(11)	0.30607(17)
			Ag	0.26675(4)	0.26666(4)	0.26693(5)
			Sb	0.25409(12)	0.25399(10)	0.25429(15)

Sr_2ZnSb_2

T (K)	R_{wp} (%)	χ^2	Atom	B_{eq} (Å^2)	$B_{11} = B_{22} = 2B_{12}$ (Å^2)	B_{33} (Å^2)
10	3.88	1.40	Sr	0.37707(4)	0.43707(3)	0.25707(5)
			Zn	0.08150(10)	0.00349(8)	0.23752(13)
			Sb	0.13427(5)	0.06296(4)	0.27690(7)
50	3.85	1.37	Sr	0.42944(5)	0.49819(3)	0.29194(10)
			Zn	0.09785(5)	0.00438(4)	0.28478(8)
			Sb	0.02327(3)	0.09565(2)	0.32911(4)
100	3.83	1.37	Sr	0.53956(5)	0.61956(5)	0.37956(7)
			Zn	0.12671(10)	0.01278(8)	0.35455(15)
			Sb	0.24158(8)	0.15235(6)	0.42005(13)

			Sr	0.65663(8)	0.75797(3)	0.45397(17)
150	3.82	1.37	Zn	0.20915(8)	0.01659(7)	0.59426(11)
			Sb	0.34259(6)	0.18921(2)	0.64933(14)
			Sr	0.87260(7)	0.98034(6)	0.65714(9)
200	3.86	1.41	Zn	0.30272(7)	0.02195(6)	0.86427(9)
			Sb	0.46657(11)	0.21919(9)	0.96131(14)
			Sr	1.11601(4)	1.23338(3)	0.88127(5)
250	3.76	1.36	Zn	0.39880(4)	0.03557(3)	1.12525(5)
			Sb	0.62837(12)	0.31870(8)	1.24770(19)
			Sr	1.27142(6)	1.44040(5)	0.93345(7)
300	3.72	1.34	Zn	0.49936(11)	0.05551(8)	1.38706(17)
			Sb	0.80844(8)	0.45760(6)	1.51014(13)

The data analyses were performed on the basis of B_{ov} refinement result, and only B_{aniso} was refined while other parameters were fixed. The data are extracted from the Rietveld refinement of the powder neutron diffraction data obtained from SuperHRPD diffractometer. B_{eq} is the equivalent ADPs as determined by the formula of $B_{\mathrm{eq}} = \frac{1}{3}(B_{11} + B_{22} + B_{33})$.

Supplementary references

1. Willis, B. T. M. & Pryor, A. W. *Thermal Vibrations in Crystallography Ch. 4* (Cambridge University Press, London, 1975).

2. Sevillano, E., Meuth, H. & Rehr, J. J. Extended x-ray absorption fine structure Debye-Waller factors. I. Monatomic crystals. *Phys. Rev. B* **20**, 4908-4911 (1979).

3. Kurosaki, K., *et al.* Ag_9TlTe_5: A high-performance thermoelectric bulk material with extremely low thermal conductivity. *Appl. Phys. Lett.* **87**, 061919 (2005).

4. Xiao, Y., *et al.* Origin of low thermal conductivity in SnSe. *Phys. Rev. B* **94**, 125203 (2016).

5. Lin, Y., Islam, F. & Kresh, M. Multiphonon: Phonon density of states tools for inelastic neutron scattering powder data. *J. Open Source Softw.* **3**, 440 (2018).

6. Li, W., *et al.* ShengBTE: A solver of the Boltzmann transport equation for phonons. *Comput. Phys. Commun.* **185**, 1747-1758 (2014).

7. Wang, C., *et al.* Intrinsic Zn Vacancies-Induced Wavelike Tunneling of Phonons and Ultralow Lattice Thermal Conductivity in Zintl Phase Sr_2ZnSb_2. *Chem. Mater.* **34**, 7837-7844 (2022).

8. Ratsifaritana, C. A. & Klemens, P. G. Scattering of phonons by vacancies. *Int. J. Thermophys.* **8**, 737-750 (1987).

9. Tan, G., *et al.* High Thermoelectric Performance in SnTe–$AgSbTe_2$ Alloys from Lattice Softening, Giant Phonon–Vacancy Scattering, and Valence Band Convergence. *ACS Energy Lett.* **3**, 705-712 (2018).

10. Slade, T. J., *et al.* Contrasting SnTe–$NaSbTe_2$ and SnTe–$NaBiTe_2$ Thermoelectric Alloys: High Performance Facilitated by Increased Cation Vacancies and Lattice Softening. *J. Am. Chem. Soc.* **142**, 12524-12535 (2020).
